# Supplementary material for: Association between oral microbiome and seven types of cancers in East Asian population: a two-sample Mendelian randomization analysis
Source: Front Mol Biosci. 2023 Nov 21;10:1327893. doi: 10.3389/fmolb.2023.1327893 (PMC10702768; doi:10.3389/fmolb.2023.1327893)
Supplement: Supplementary file 1 [file DataSheet1.ZIP › Supplementary Materials/MR plots for tongue/tongue═╝/Lung cancer/pheno.3163_to_lung cancer_forest.pdf]

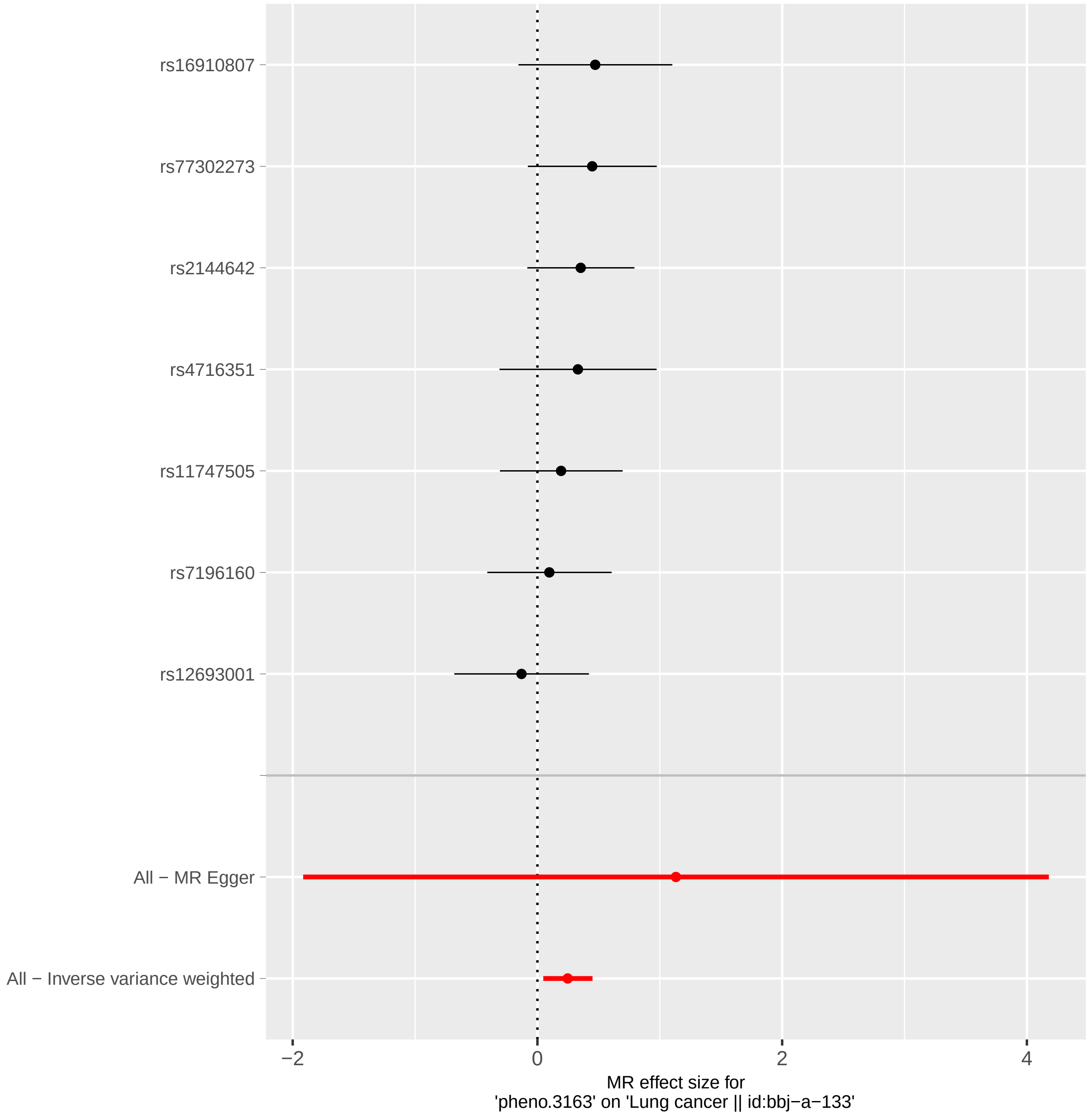
rs16910807
rs77302273
rs2144642
rs4716351
rs11747505
rs7196160
rs12693001
All − MR Egger
All − Inverse variance weighted
−2
0
2
4
MR effect size for
'pheno.3163' on 'Lung cancer || id:bbj−a−133'
